# Supplementary material for: Unveiling the Bioactive Potential of the Invasive Jellyfish Phyllorhiza punctata Through Integrative Transcriptomic and Proteomic Analyses
Source: Biomolecules. 2025 Aug 4;15(8):1121. doi: 10.3390/biom15081121 (PMC12383608; doi:10.3390/biom15081121)
Supplement: Supplementary file 1 [file biomolecules-15-01121-s001.zip › Suplementary Material/FileS3/PEPFOLD4/XP_065060541_515/clusters.html]

 PEP-FOLD clusters


Download archive of all models  here   

|  |  |  |
| --- | --- | --- |
| # | file | sOPEP |
| # cluster 1 - Size: 23 |  |  |
|  | XP\_065060541\_515-model1 | -9.96672 |
|  | XP\_065060541\_515-model1.1 | -9.96672 |
|  | XP\_065060541\_515-model1.2 | -9.96672 |
|  | XP\_065060541\_515-model1.3 | -9.96672 |
|  | XP\_065060541\_515-model1.4 | -9.96672 |
|  | XP\_065060541\_515-model1.5 | -9.96672 |
|  | XP\_065060541\_515-model1.6 | -9.96672 |
|  | XP\_065060541\_515-model1.7 | -9.96672 |
|  | XP\_065060541\_515-model1.8 | -9.96672 |
|  | XP\_065060541\_515-model1.9 | -9.96672 |
|  | XP\_065060541\_515-model1.10 | -9.96672 |
|  | XP\_065060541\_515-model1.11 | -9.96672 |
|  | XP\_065060541\_515-model1.12 | -9.96672 |
|  | XP\_065060541\_515-model1.13 | -9.96672 |
|  | XP\_065060541\_515-model1.14 | -9.96672 |
|  | XP\_065060541\_515-model1.15 | -9.96672 |
|  | XP\_065060541\_515-model1.16 | -9.85981 |
|  | XP\_065060541\_515-model1.17 | -9.85981 |
|  | XP\_065060541\_515-model1.18 | -9.84741 |
|  | XP\_065060541\_515-model1.19 | -9.84741 |
|  | XP\_065060541\_515-model1.20 | -9.84741 |
|  | XP\_065060541\_515-model1.21 | -9.84741 |
|  | XP\_065060541\_515-model1.22 | -9.83714 |
| # cluster 2 - Size: 14 |  |  |
|  | XP\_065060541\_515-model2 | -4.69352 |
|  | XP\_065060541\_515-model2.1 | -4.63325 |
|  | XP\_065060541\_515-model2.2 | -4.56614 |
|  | XP\_065060541\_515-model2.3 | -4.29231 |
|  | XP\_065060541\_515-model2.4 | -4.29231 |
|  | XP\_065060541\_515-model2.5 | -4.29231 |
|  | XP\_065060541\_515-model2.6 | -4.29231 |
|  | XP\_065060541\_515-model2.7 | -4.25705 |
|  | XP\_065060541\_515-model2.8 | -4.25705 |
|  | XP\_065060541\_515-model2.9 | -4.20091 |
|  | XP\_065060541\_515-model2.10 | -4.18172 |
|  | XP\_065060541\_515-model2.11 | -4.17884 |
|  | XP\_065060541\_515-model2.12 | -4.1657 |
|  | XP\_065060541\_515-model2.13 | -4.06115 |
| # cluster 3 - Size: 4 |  |  |
|  | XP\_065060541\_515-model3 | -4.56868 |
|  | XP\_065060541\_515-model3.1 | -4.56868 |
|  | XP\_065060541\_515-model3.2 | -4.26412 |
|  | XP\_065060541\_515-model3.3 | -4.15182 |
| # cluster 4 - Size: 58 |  |  |
|  | XP\_065060541\_515-model4 | -4.09731 |
|  | XP\_065060541\_515-model4.1 | -4.08727 |
|  | XP\_065060541\_515-model4.2 | -4.08049 |
|  | XP\_065060541\_515-model4.3 | -4.08032 |
|  | XP\_065060541\_515-model4.4 | -4.07946 |
|  | XP\_065060541\_515-model4.5 | -4.07538 |
|  | XP\_065060541\_515-model4.6 | -4.07212 |
|  | XP\_065060541\_515-model4.7 | -4.07212 |
|  | XP\_065060541\_515-model4.8 | -4.07212 |
|  | XP\_065060541\_515-model4.9 | -4.07212 |
|  | XP\_065060541\_515-model4.10 | -4.07212 |
|  | XP\_065060541\_515-model4.11 | -4.07212 |
|  | XP\_065060541\_515-model4.12 | -4.07212 |
|  | XP\_065060541\_515-model4.13 | -4.07212 |
|  | XP\_065060541\_515-model4.14 | -4.06719 |
|  | XP\_065060541\_515-model4.15 | -4.06719 |
|  | XP\_065060541\_515-model4.16 | -4.06719 |
|  | XP\_065060541\_515-model4.17 | -4.06719 |
|  | XP\_065060541\_515-model4.18 | -4.06719 |
|  | XP\_065060541\_515-model4.19 | -4.06719 |
|  | XP\_065060541\_515-model4.20 | -4.06423 |
|  | XP\_065060541\_515-model4.21 | -4.06423 |
|  | XP\_065060541\_515-model4.22 | -4.06423 |
|  | XP\_065060541\_515-model4.23 | -4.05873 |
|  | XP\_065060541\_515-model4.24 | -4.0559 |
|  | XP\_065060541\_515-model4.25 | -4.05495 |
|  | XP\_065060541\_515-model4.26 | -4.05374 |
|  | XP\_065060541\_515-model4.27 | -4.05374 |
|  | XP\_065060541\_515-model4.28 | -4.05374 |
|  | XP\_065060541\_515-model4.29 | -4.04998 |
|  | XP\_065060541\_515-model4.30 | -4.04998 |
|  | XP\_065060541\_515-model4.31 | -4.04998 |
|  | XP\_065060541\_515-model4.32 | -4.04998 |
|  | XP\_065060541\_515-model4.33 | -4.04998 |
|  | XP\_065060541\_515-model4.34 | -4.04932 |
|  | XP\_065060541\_515-model4.35 | -4.04932 |
|  | XP\_065060541\_515-model4.36 | -4.04428 |
|  | XP\_065060541\_515-model4.37 | -4.04428 |
|  | XP\_065060541\_515-model4.38 | -4.04428 |
|  | XP\_065060541\_515-model4.39 | -4.0325 |
|  | XP\_065060541\_515-model4.40 | -4.031 |
|  | XP\_065060541\_515-model4.41 | -4.031 |
|  | XP\_065060541\_515-model4.42 | -4.03029 |
|  | XP\_065060541\_515-model4.43 | -4.03029 |
|  | XP\_065060541\_515-model4.44 | -4.02988 |
|  | XP\_065060541\_515-model4.45 | -4.02895 |
|  | XP\_065060541\_515-model4.46 | -4.02843 |
|  | XP\_065060541\_515-model4.47 | -4.02843 |
|  | XP\_065060541\_515-model4.48 | -4.02723 |
|  | XP\_065060541\_515-model4.49 | -4.02723 |
|  | XP\_065060541\_515-model4.50 | -4.02437 |
|  | XP\_065060541\_515-model4.51 | -4.02437 |
|  | XP\_065060541\_515-model4.52 | -4.02437 |
|  | XP\_065060541\_515-model4.53 | -4.02326 |
|  | XP\_065060541\_515-model4.54 | -4.02164 |
|  | XP\_065060541\_515-model4.55 | -4.02164 |
|  | XP\_065060541\_515-model4.56 | -4.01528 |
|  | XP\_065060541\_515-model4.57 | -4.00813 |
| # cluster 5 - Size: 1 |  |  |
|  | XP\_065060541\_515-model5 | -3.99415 |
|  |  |  |
